# Early maternal separation accelerates the progression of endometriosis in adult mice

**DOI:** 10.1186/s12958-020-00600-4

**Published:** 2020-06-12

**Authors:** Qiqi Long, Xishi Liu, Sun-Wei Guo

**Affiliations:** 1 Shanghai Obstetrics and Gynecology Hospital, Fudan University, Shanghai, 200090 China; 2 Shanghai Key Laboratory of Female Reproductive Endocrine-Related Diseases, Fudan University, Shanghai, China; 3 Shanghai Obstetrics and Gynecology Hospital, Fudan University Shanghai College of Medicine, 419 Fangxie Road, Shanghai, 200011 China

**Keywords:** Adrenergic receptor, Early life adversity, Endometriosis, Maternal separation, Mouse

## Abstract

**Background:**

A large body of research highlights the importance of early-life environmental impact on the health outcome in adulthood. However, whether early-life adversity (ELA) has any impact on the development of endometriosis is completely unclear. In this study, we tested the hypothesis that ELA, as manifested by neonatal separation, can accelerate the progression of endometriosis in mouse through activation of the adrenergic receptor β2 (ADRB2) signaling pathway, leading to increased angiogenesis and progression of endometriotic lesions.

**Methods:**

Eight female Balb/C mice, in late pregnancy, were used used for this study, which later gave birth to 22 female newborn pubs. Eleven additional female Balb/C mice were also used as donors of uterine tissues. The 22 newborn pubs were randomly divided into 2 equal-sized groups, maternal separation (MS) and no separation (NS). Pubs in the MS group were separated from their dams for 3 h/day from postnatal day (PND) 1 to 21, while those in the NS control remained in the home cage with their dams. In adulthood (8-week old), 3 mice in each group were randomly selected to undergo a battery of behavior tests. The remaining 8 mice in each group were induced with endometriosis by intraperitoneal injection of uterine fragments from donor mice. Four weeks after the induction, all mice were sacrificed and their endometriotic lesions were excised for quantification and then prepared for immunohistochemistry analysis.

**Results:**

We confirmed that MS during infancy resulted in anxiety and depression-like behaviors as previously reported. We also found that in MS mice the lesion weight was increased by over 2 folds and generalized hyperalgesia was also significantly increased as compared with NS mice. Immunostaining analysis demonstrated that MS accelerated the development of endometriosis likely through decreased dopamine receptor D2 (DRD2) expression and activation of the ADRB2/cAMP-response element binding protein (CREB) signaling pathway, leading to increased angiogenesis and progression of endometriotic lesions.

**Conclusions:**

Exposure of female mouse pups to ELA such as MS during their infancy period accelerates the progression of endometriosis, possibly through altered neuronal wiring and hyperactivity of the hypothalamic-pituitary-adrenal axis.

## Introduction

Endometriosis, characterized by the ectopic presence and growth of functioning endometrial tissues, is a disease affecting 6–10% of women of reproductive age [1]. Despite its high prevalence and debilitating nature impacting negatively on the quality of life in women afflicted with the disease [2], its pathogenesis is still poorly understood, and few, if any, modifiable risk factors have been identified [3].

In the last three decades, a large body of research highlights the importance of early-life environmental impact on the health outcome in adulthood. Notably, the hypothesis of “developmental origin of adult health and disease” (DOHaD) was proposed, which posits that unfavorable environmental early-life conditions can lead to “developmental programming” in later life and the subsequent modification in risks of many chronic diseases [4–6]. Initially concerned with the impact of prenatal and neonatal malnutrition on adult health, the hypothesis has received empirical support from epidemiological studies worldwide. Over the years, however, it became evident that non-nutritional, early-life adversity (ELA), such as abuse, neglect and traumatic events, also can have long-lasting effect and profoundly impact on the health status throughout adult life, likely mediated through epigenetic mechanisms [7, 8]. These poor health outcomes not only include psychological disorders [9, 10] but also several somatic pathologies [11, 12].

Very few research on the early-life environment effect on the risk of endometriosis has been published. Intrauterine exposure to diethylstilbestrol is reported to be associated with elevated risk of endometriosis [13], while intrauterine exposure to smoking is reported to be associated with the reduced risk [14]. Preterm birth, formula feeding [15], and regularly fed soy formula in infancy [16] are found to increase the risk. Only one study reported, somewhat tangentially, the link between non-nutritional ELA and endometriosis: childhood physical abuse is reported to be associated with endometriosis [17].

The scarcity of such research is attributable, perhaps in no small amount, to various challenges in conducting such studies. However, exploring the impact of ELA using animal models may be feasible, and the resultant outcome may help us to gain better understanding on such an impact, if any, on endometriosis and perhaps also gain much needed mechanistic insight, which is otherwise difficult for human studies.

A growing body of evidence indicates that ELA, such as physical or sexual abuse, neglect, and loss of a parent, exerts its long-term detrimental effect on health through sensitization or enhanced vulnerability to subsequent stressors, likely involving dysregulated stress response [18]. One such stress response system as a possible mechanism underlying stress sensitization is the hypothalamic-pituitary-adrenal (HPA) axis, which undergoes protracted development throughout childhood and adolescence and may be particularly vulnerable to ELA [19].

Early maternal separation (MS), an ELA animal model, has been widely used to investigate prolonged effects of ELA on neurobiological and behavioral outcomes in adulthood [20]. Prolonged MS results in the hyper-reactivity of the HPA axis in adulthood and lasts for life [20]. MS in rodents also induces a greater susceptibility of the HPA axis to acute stress in adults [21, 22]. Besides depressive-like behavior, MS can lead to anxiety-like behavior and cognitive deficits in adulthood [23], which are known to be stressors.

We and others have previously reported that chronic stress promotes the development of endometriosis in mice [24–26]. This promotional effect is likely to be mediated through the deregulated HPA axis and the systemic activation of the sympatho-adreno-medullary (SAM) axis, which results in subsequent secretion of catecholamines. The surging catecholamines may activate the adrenergic receptor β2 (ADRB2) and cAMP-response element binding protein (CREB) in endometriotic lesions, yielding increased angiogenesis and proliferation in mice with induced endometriosis [24]. In addition, chronic psychogenic stress also induces epigenetic changes in the hippocampus in mice with endometriosis, aside from lesional activation of ADRB2/CREB signaling pathway, facilitating the progression of endometriosis [27].

In this study, we hypothesized that ELA, as manifested by MS, can accelerate the progression of endometriosis in mouse, likely through increased anxiety and depression levels. This study was undertaken to test this hypothesis.

## Materials and methods

### Animals

Eight female Balb/C mice, in late gestation stage, were purchased from the SLAC Experimental Animal Company (Shanghai, China) and used for this study. Three days after arriving at the experimental animal facility, all mice delivered. Only female newborn pubs, totaling 22, were used in the experiment. These pubs were divided into 2 parts, one with 6 and the other, 16. The 6 pubs were used for the validation experiment that MS can induce anxiety and depression like behaviors in adulthood (8 weeks of age). The remaining 16 pubs were used to see whether MS can accelerate the progression of endometriosis. Eleven additional female Balb/C mice, of 7 weeks old, were purchased as donors from the same company. All mice were maintained under controlled conditions with a light/dark cycle of 12/12 h and had access to food and water ad libitum. All experiments were performed under the guidelines of the National Research Council’s *Guide for the Care and Use of Laboratory Animals* [28] and approved by the institutional experimental animals review board of Shanghai OB/GYN Hospital, Fudan University.

### Maternal separation and experimental design

Twenty-two newborn female Balb/C pubs were randomly divided into two equal-sized groups: the non-separated (NS) group and the MS group. Designating the day of delivery as postnatal day (PND) 0, pups in the MS group were separated from their respective dams for 3 h daily from PND 1 to PND 21 [29]. The pups were then returned to their mothers after 3 h of MS. In the NS group, the pups were left with their respective dams undisturbed, as usual [29]. At PND 21, the pups were weaned and transferred to new cages with 3–5 animals per cage. They were left undisturbed, and were under routine animal care [29]. In adulthood (8-week old), 3 mice in each group were randomly selected for evaluating the levels of depression and anxiety via several behavioral tests, including forced swimming test, tail suspension test and open field test. The remaining 8 mice in each group procedure to induce endometriosis through injection of uterine fragments. Four weeks after the induction, all mice were sacrificed and their endometriotic lesions were excised for quantification and then prepared for immunohistochemistry analysis. Before the induction and sacrifice, hotplate test was carried out.

### Induction of endometriosis

We used an established mouse model of endometriosis by intraperitoneal (i.p.) injection of endometrial fragments as described [30–32] and also used in our previous studies [24, 33]. Briefly, donor mice were initially injected with 100 μg/kg estradiol benzoate (Animal Medicine Factory, Hangzhou, China). One week later they were sacrificed and their uteri were removed and harvested. The uterine tissues were seeded in a Petri dish containing warm sterile saline, and split longitudinally with a pair of scissors.

Two uterine horns from each mouse were first minced with scissors, ensuring that the maximal diameter of the fragment was consistently smaller than 1 mm. Then uterine fragments were intraperitoneally injected to recipient mice. Thus each mouse received the suspension derived from a half uterus. To eliminate any potential bias, endometrial fragments from 1 donor mice were mixed together and injected i.p. to 2 mice, one each from the two groups. By this approach, any individual variation was minimized.

### Behavior tests

The forced swimming test (FST) and tail suspension test (TST) were performed to measure depression related behaviors in rodents. The open field test (OFT) was performed to measure anxiety-related behaviors.

#### Forced swimming test (FST)

The procedure was carried out following previously reported procedures [34, 35]. Each mouse was placed individually into glass cylinders (height 25 cm, diameter 10 cm) containing 10 cm of water at 23-25 °C. The animals were left in the cylinder for 6 min. The total duration of immobility was recorded during the last 4 min of the 6-min long testing period. The mouse was judged to be immobile when it ceased struggling and remained floating motionless in the water, making only those movements necessary to keep its head above the water level. The immobility time was scored in real time by two observers who were blinded to the grouping. The results obtained in FST were presented as the arithmetic mean of the immobility time of animals given in seconds for each experimental group.

#### Tail suspension test (TST)

The procedure was carried out according to the method as described previously [34, 36]. In this test, the mouse was suspended from its tail for a fixed time period and its movements were recorded. The ratio between immobility and agitation determines the depressive state of the animal [36].

Briefly, each mouse was individually suspended by the tail to a vertical bar in a wooden box (30 * 30 cm). The animals were fastened by means adhesive tape fixed 2 cm from the end of the tail for 6 min. The total duration of immobility was recorded during the last 4 min of the 6-min long testing period. The mouse was judged to be immobile when it ceased moving its limbs and body, making only those movements necessary to breathe. The immobility time was scored in real time by two observers who were blinded to the grouping. The results obtained in TST were presented as the arithmetic mean of the immobility time of animals given in seconds for each experimental group.

#### Open field test

The procedure was carried out according to the method as described previously [37]. Mice were placed into the center of a Plexiglas box (50 cm × 50 cm × 40 cm) in a brightly lit room. During a 5-min session, animals were scored for the number of rearing. Animal behavior was recorded and subsequently analyzed using a video-tracking system (Shanghai Mobile Datum Information Technology Company, Shanghai, China).

### Hotplate test

The hotplate test was performed with a commercially available Hot Plate Analgesia Meter (Model BME-480, Institute of Biomedical Engineering, Chinese Academy of Medical Sciences, Tianjin, China) as reported previously [38]. The hotplate consisted of a metal plate of 26 cm × 26 cm, and its surface was heated to a constant temperature of 55.0 °C ± 0.1 °C. A plastic cylinder (22 cm in diameter, 12 cm in height) was placed on the hotplate. Mice were brought to the testing room and allowed to acclimatize for 10 min before the test. The latency to respond to thermal stimulus is defined as the time (in seconds) elapsed from the moment when the mouse is inserted into the cylinder until it licks its hind paws, or jolted or jumped off the hot plate. Each mouse was tested only once in each session.

### Immunohistochemistry

Tissue samples were fixed in 4% (w/v) paraformaldehyde and paraffin-embedded. Serial 4-μm sections were obtained from each block, with the first resultant slide being hematoxylin-eosin staining stained to confirm pathologic diagnosis, and the subsequent slides stained for ADRB2, dopamine D2 receptor (DRD2), α-smooth muscle actin (α-SMA), CD31 (for microvessel density or MVD), E-cadherin (for epithelial cells), as well as Masson trichrome staining.

Routine deparaffinization and rehydration procedures were performed. For antigen retrieval, the slides were heated at 98 °C in a citrate buffer (pH 6.0) for a total of 30 min for staining for α-SMA, E-cadherin, DRD2, or in an EDTA buffer (pH 8.0, Shanghai Sun BioTech Company, Shanghai, China) for a total of 20 min for staining for ADRB2, CD31, and then cooled naturally to the room temperature. The primary antibodies against ADRB2, DRD2, α-SMA, CD31, E-cadherin were diluted to 1:50, 0.5 μg/ml, 1:100, 1:50, 1:400, respectively, and sections were incubated with the primary antibody overnight at 4 °C. After slides were rinsed, the HRP labeled secondary antibody Detection Reagent (Sunpoly-HII, BioSun Technology Co., Ltd., Shanghai, China) was incubated at room temperature for 30 min. The bound antibody complexes were stained for 3–5 min or until appropriate for microscopic examination with diaminobenzidine and then counterstained with hematoxylin (30 s) and mounted. The names of primary antibodies, along with their vendor names and the concentrations used in this study are listed in Table 1.

**Table 1:** List of names and catalog numbers of antibodies used in this study

Antibody name	Company	Catalog number	Concentration
ADRB2	Abcam	ab61778	1:50
DRD2	Thermo Fisher	PA5-79169	0.5 μg/ml
α-SMA	Abcam	ab5694	1:100
CD31	Abcam	ab28364	1: 50
E-cadherin	CST	#3195	1:400

Images were obtained with the microscope (Olympus BX51, Olympus, Tokyo, Japan) fitted with a digital camera (Olympus DP70, Olympus). For other immunostained markers, quantification was made through 3-5 randomly selected images for each mouse at 400X magnification were taken to obtain a mean optional density value by Image Pro-Plus 6.0 (Media Cybernetics, Inc., Bethesda, MA, USA), as reported previously.

Human brain, mouse brain, mouse liver tissue and human breast cancer were used as positive controls for ADRB2, DRD2, α-SMA, CD31, E-cadherin. For negative controls, mouse ectopic endometrium tissue samples were incubated with rabbit or mouse serum instead of primary antibodies.

To minimize potential bias, the person who evaluated the slides was blinded as to which group the slides belonged to.

### Masson trichrome staining

For Masson trichrome staining, tissue sections were deparaffinized in xylene and rehydrated in a graded alcohol series and then were immersed in Bouin solution at 37 °C for 2 h. Bouin solution was made with 75 mL of saturated picric acid, 25 mL of 10% formalin (w/v) solution, and 5 mL of acetic acid. Tissue sections were stained using the Masson Trichrome Staining kit (Baso, Wuhan, China) following the manufacturer’s instructions. The areas of the collagen fiber layer stained in blue in proportion to the entire field of the ectopic implants were calculated by the Image Pro-Plus 6.0 (Media Cybernetics).

### Statistical analysis

The comparison of distributions of all three behavior tests between the two groups was made using the two-group comparison Student’s t-test. Since the sample size was small (*n* = 3 in each group) and the normality assumption was unlikely to hold, a permutation test was also used based on 10^6^ permutations. The two-group comparison for hotplate latency and immunostaining levels was made using Wilcoxon’s test. *P* values of less than 0.05 were considered statistically significant. All computations were made with R 3.6.2 [39].

## Results

### Neonatal maternal separation elevates anxiety and depression levels in adult mice

**Fig. 1:**
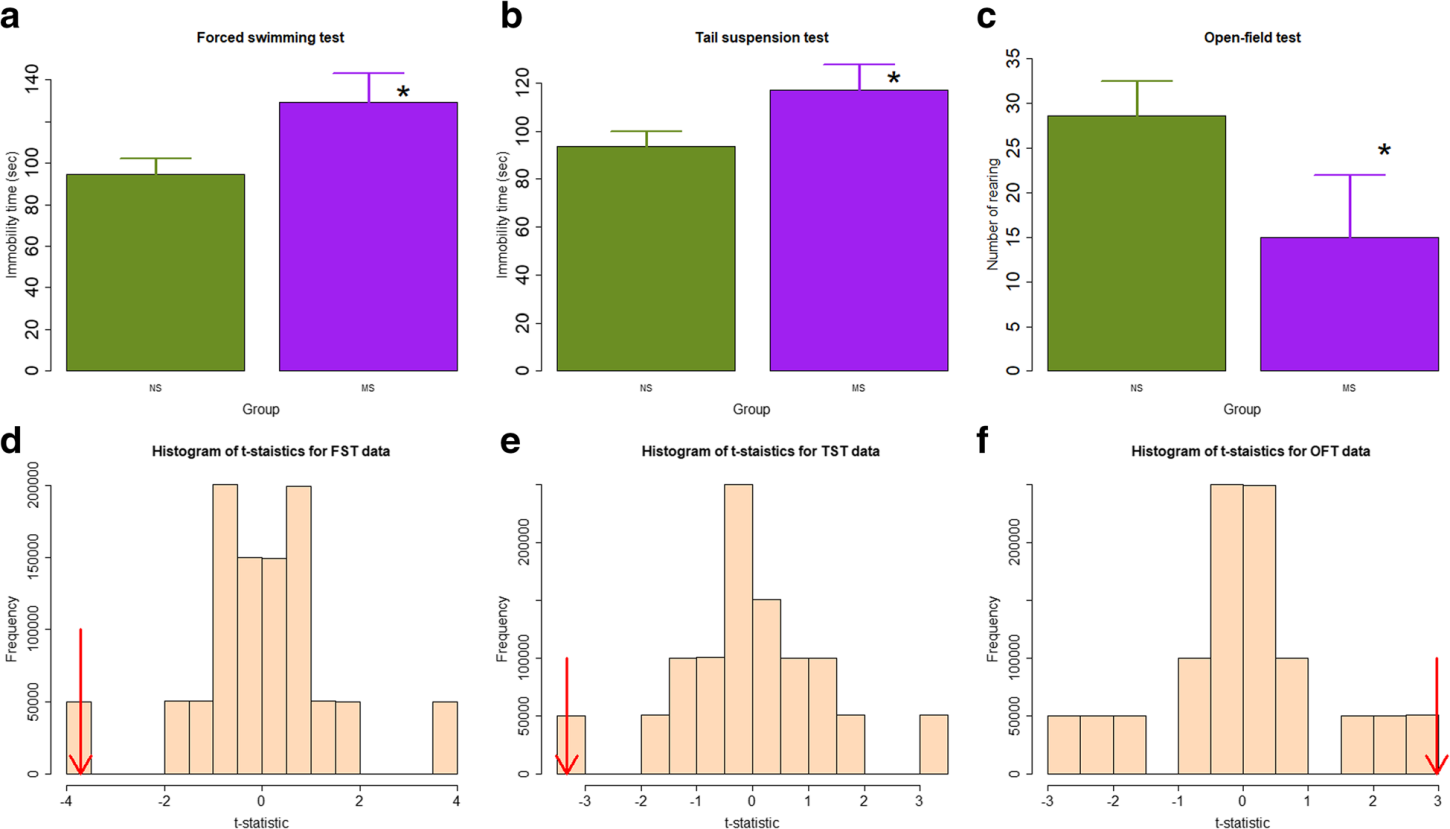
Summary of behavioral test results. **a** Difference in mean immobility time of the forced swimming test; **b** Mean immobility time of the tail suspension test, and **c** Average rearing number of mice in the open field test between the two groups. n = 3 in each group. NS: non-separated; MS: maternal separation. Data are presented in mean ± SD. Symbols for the statistical significance level: *: *p* < 0.05; #: *p* = 0.057. Permutation test results, based on one million permutations, for the t-statistics for the data of forced swimming test (**d**), tail suspension test (**e**), and open field test (**f**). The red arrows indicate the calculated t-statistic for the original data

We first evaluated as whether there is any behavioral change in the adult mice exposed to MS, and found significant changes. Specifically, in the forced swimming test, the mean immobility time of mice in the MS group was significantly longer than that of the NS mice (129.0 ± 14.2 vs. 94.7 ± 7.4 s, *p* = 0.034; Fig. 1a). Consistently, in the tail suspension test, the mean immobility time of MS mice was also significantly longer than the NS mice (117.0 ± 10.5 vs. 93.7 ± 6.0 s, *p* = 0.041; Fig. 1b). In contrast, in the open field test, the average rearing number of the MS mice was marginally significantly less than that of NS mice (28.7 ± 7.0 vs. 15.0 ± 3.8, *p* = 0.057; Fig. 1c). Permutation test indicated that the results were all statistically significant (all *p*-values < 0.05; Fig. 1d-f). Taken together, these data collectively indicate that mice in the MS group exhibit more signs of depression as compared with NS mice.

### MS promotes the development of endometriosis and exacerbates generalized hyperalgesia in adult mice

**Fig. 2:**
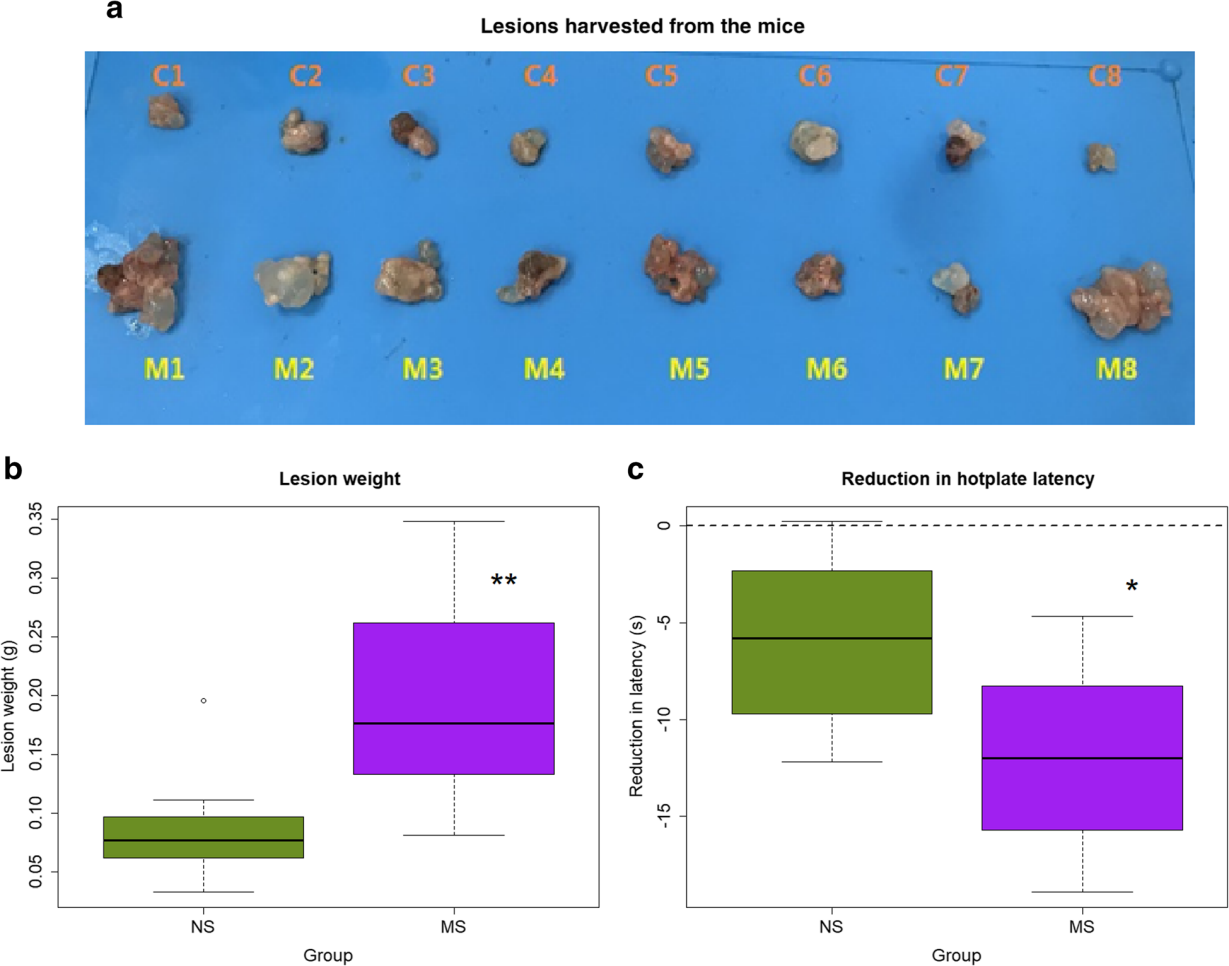
The effect of early maternal separation on endometriotic lesions and generalized hyperalgesia. **a** Photographs of excised lesions in mice in the NS and MS groups. C: lesions taken from NS mouse; M: lesions taken from MS mouse. **b** Boxplot of total lesion weight in NS and MS mice; **c** Reduction in the mean hotplate latency relative to the baseline levels. The dashed line represents no change. *n* = 8 in each group. NS: non-separated; MS: maternal separation. Symbols for the statistical significance level: *: p < 0.05; **: *p* < 0.01

We found that lesions harvested from the mice in the MS group were conspicuously larger than that from NS mice (Fig. 2a), and the total lesion weight was significantly increased as compared with the NS mice (*p* < 0.007; Fig. 2b). In fact, the mean lesion weight in MS mice increased more than 2 folds as compared with NS mice (196.4 ± 87.5 mg vs 87.6 ± 49.0 mg, or an increase by 124.3%).

There was no statistically significant difference in hotplate latency between the MS and NS mice before the induction of endometriosis (*p* > 0.05). Four weeks after the induction, the hotplate latency was significantly reduced as the result of endometriosis (*p* = 6.1 × 10^− 5^). However, mice in the MS group had significantly more reduction in latency as compared with that of NS mice (*p* = 0.0499; Fig. 2c).

### MS increases ADRB2 staining, microvessel density and the extent of lesional fibrosis

**Fig. 3:**
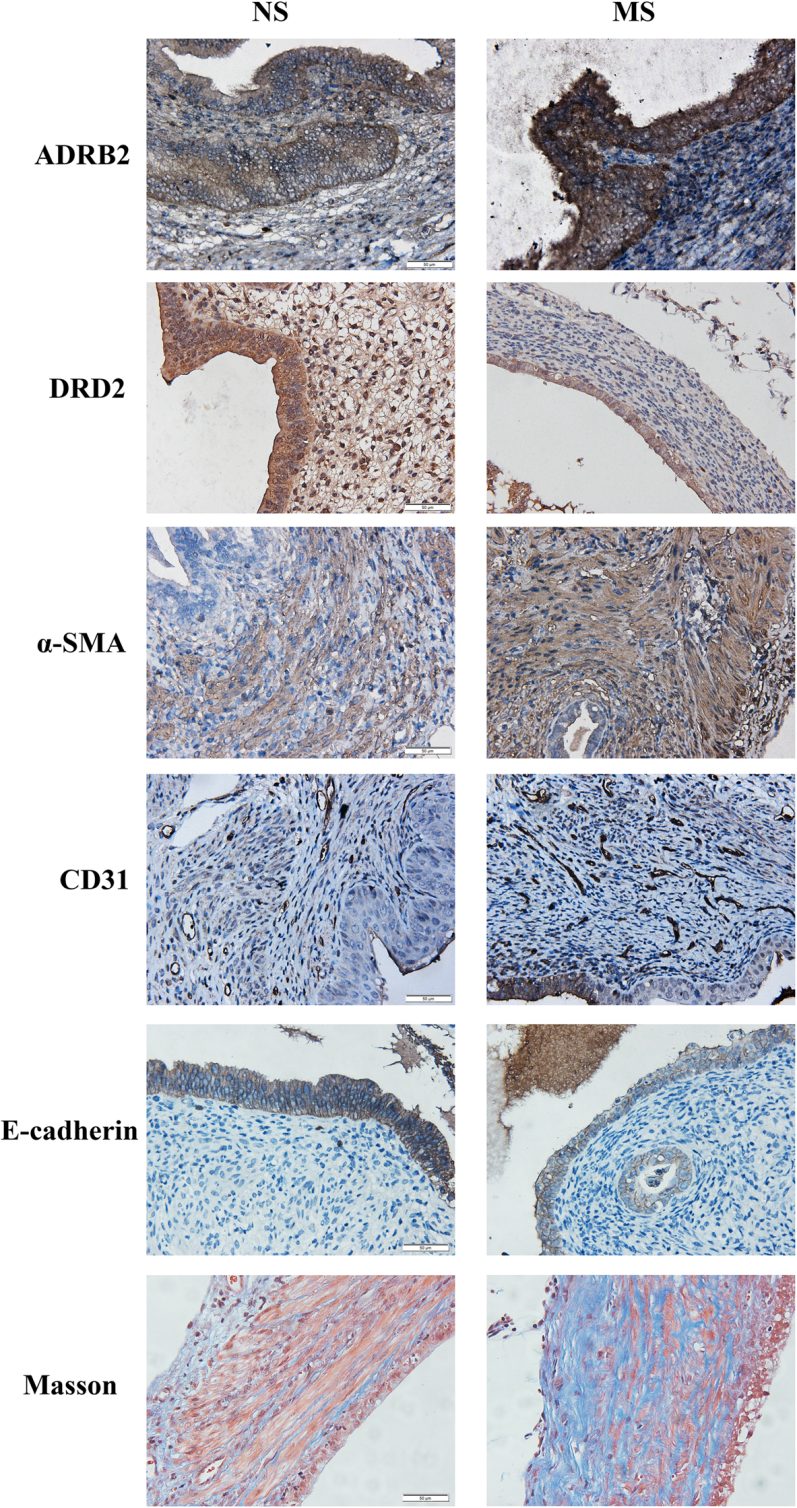
Representative immunohistochemical staining of ADRB2, DRD2, α-SMA, CD31, E-cadherin and Masson trichrome staining. The columns are for NS and MS groups, while the rows are for ADRB2, DRD2, α-SMA, CD31, E-cadherin and Masson trichrome staining, respectively. *n* = 8 in each group. NS: non-separated; MS: maternal separation. All magnifications: × 400. Scale bar = 50 μm

We next examined immunoreactivity against ADRB2, DRD2, α-SMA, CD31-stained MVD, E-cadherin, along with the extent of fibrosis via Masson trichrome staining, in all lesions from mouse with induced endometriosis. As shown in Fig. 3, ADRB2, DRD2 and E-cadherin immunoreactivity was seen primarily in glandular epithelial cells and was localized in the cytoplasm. Aside from smooth muscle cells, α-SMA staining was also observed in the cytoplasm of both epithelial cells and stromal cells. In contrast, CD31 immunostaining was seen mostly in vascular endothelial cells (Fig. 3).

**Fig. 4:**
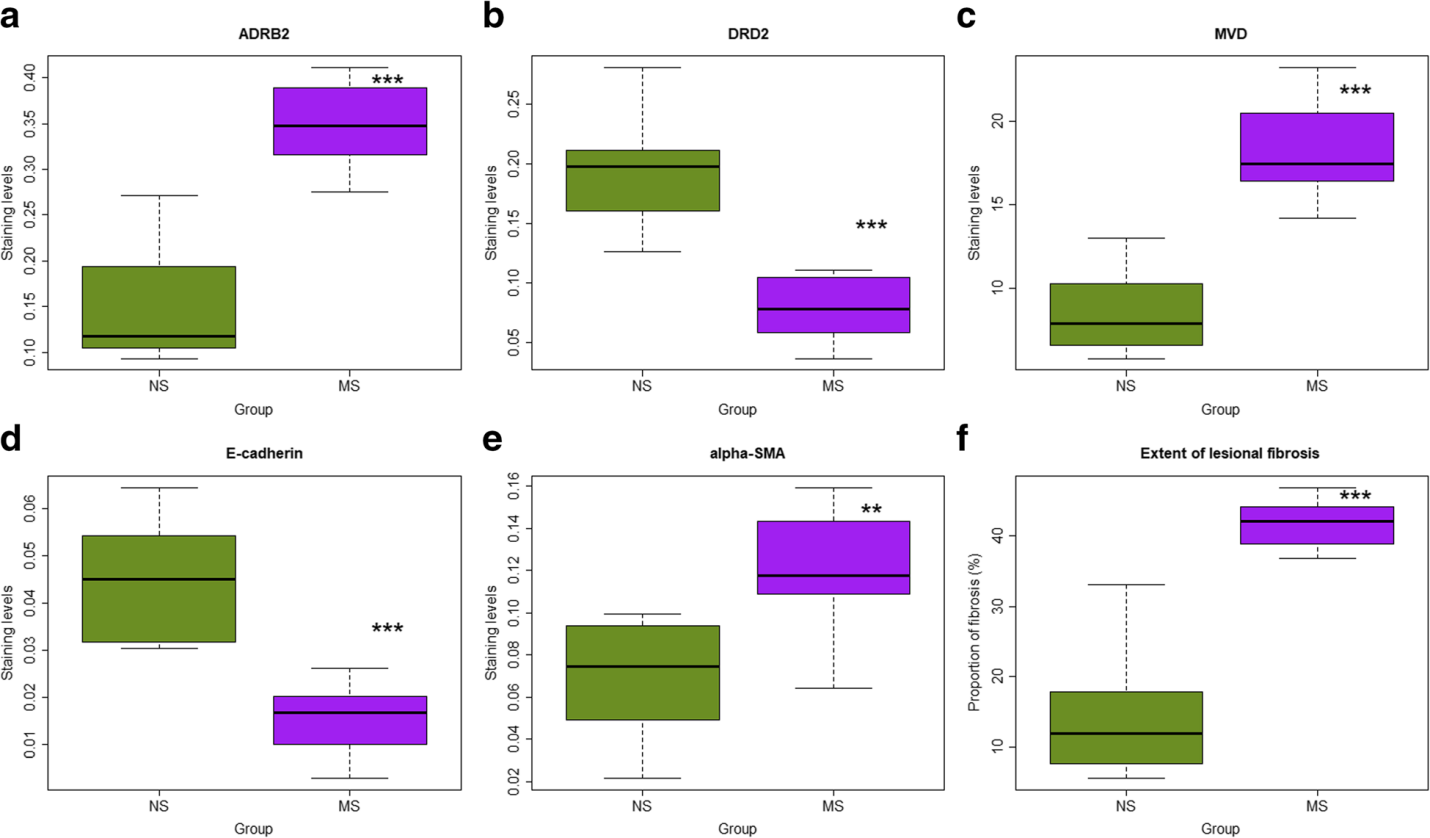
Boxplots summarizing the immunostaining results. **a** ADRB2; **b**, DRD2; **c** α-SMA; **d** CD31-positve microvessel density (MVD); **e** E-cadherin and **f** the extent of lesional fibrosis as evaluated by Masson trichrome staining in endometriotic lesions in two groups of mice. n = 8 in each group. NS: non-separated; MS: maternal separation. Symbols for the statistical significance level: *: p < 0.05; **: p < 0.01; ***: *p* < 0.001

We found that MS resulted in significantly higher staining of ADRB2 and α-SMA (*p* = 0.0002, and *p* = 0.0019, respectively; Fig. 4a,e) but significantly lower staining of DRD2 and E-cadherin (both p’s = 0.0002; Fig. 4b,d) in ectopic endometrium as compared with NS mice. Consistent with the increased lesion size, MS yielded significantly higher MVD (*p* = 0.0009; Fig. 4c) and more fibrotic content (p = 0.0002; Fig. 4f).

The lesional ADRB2 and DRD2 staining levels were closely correlated (r = − 0.76, *p* = 0.0006). Both were correlated closely with the lesional MVD (r = 0.81, *p* = 0.0001, and r = − 0.68, *p* = 0.004, respectively). While the lesion weight correlated marginally with the reduction in hotplate latency (r = − 0.50, *p* = 0.055), the extent of lesional fibrosis correlated significantly (r = − 0.55, *p* = 0.029).

## Discussion

In this study, we first confirmed that MS during infancy results in anxiety and depression-like behaviors in mouse, just as reported extensively [40, 41]. In addition, we have shown that MS promotes the development of endometriosis and exacerbates endometriosis-associated generalized hyperalgesia in adult female mice, likely through decreased DRD2 expression and activation of the ADRB2/CREB signaling pathway, leading to increased angiogenesis and progression of endometriotic lesions.

Severe adversity induced psychological stresses, such as bereavement, in early life have been reported to be associated with accelerated development of certain malignancies [42, 43]. Although such data are lacking for endometriosis, one study has reported that childhood physical abuse is associated with risk of developing endometriosis [17], suggesting that ELA-accelerated lesional progression is biologically plausible, especially given that retrograde menstruation is nearly universal and is likely the cause for endometriosis [44]. Thus, a recent proposition that trauma, physical or otherwise, increases the risk of endometriosis [45] may have some merits. Unfortunately, the impact of non-nutritional ELA on the risk of endometriosis is essentially undocumented and warrants more research. By demonstrating that MS, as a form of ELA, can accelerate the progression of endometriosis, this study provided, to our best knowledge, the first piece of experimental evidence for the promotional effect of ELA on the development of endometriosis in adulthood.

Our study is consistent with the reports that MS results in significantly higher levels of anxiety and depression [46, 47]. Anxiety and depression are both potent stressors. The increased levels of anxiety and depression in mice are unmistakable sign of chronic stress. Consequently, they are likely to induce systemic activation of the HPA and the SAM axes, resulting in increased release of glucocorticoids and catecholamines, inducing the activation of the ADRB2 and CREB signaling pathways and accelerating lesional progression in mice with induced endometriosis [24, 33]. In addition, endometriosis, in and by itself, can also induce pain, anxiety and depression [48], virtually establishing a feed-forward loop that further promotes lesional progression.

We have previously shown that endometriotic lesions are fundamentally wounds undergoing repeated tissue injury and repair (ReTIAR) [49, 50] due to their cardinal hallmark of cyclic bleeding [51]. As a result, the endometriotic lesions undergo epithelial-mesenchymal transition (EMT) and fibroblast-to-myofibroblast transdifferentiation (FMT), resulting in increased collagen production and ultimately fibrosis [52, 53]. In addition, endometriotic stromal cells are differentiated into smooth muscle cells (SMCs), leading to smooth muscle metaplasia (SMM) that is frequently seen in endometriotic lesions [54–57].

In mice exposed to MS, their lesions showed reduced E-cadherin staining but increased staining of α-SMA in lesions as compared with NS mice, along with more extensive fibrosis. These results indicate that endometriotic lesions in MS mice experienced more complete and thorough EMT and FMT, leading to greater fibrotic content---evidence for accelerated lesional progression.

This acceleration is likely due to the activation of the ADRB2/CREB signaling pathway. Endometriotic lesions in both mouse and humans express ADRB2 but DRD2 staining is reduced [27, 33]. Consequently, catecholamines, such as epinephrine and norepinephrine, resulting from chronic stress would activate the cAMP/protein kinase A (PKA) signaling pathway, which in turn results in the phosphorylation of the CREB family [58] that is known to be involved in steriodogenesis in endometriosis [59]. PKA may also cross-regulate NF-κB [60], which is also known to play a critical role in the development of endometriosis [61].

Given that natural killer (NK) cells are known to express adrenergic receptors, it has been suggested that the sympathetic nervous system (SNS) may impact negatively on NK cytotoxicity and thus tumor metastasis [62–64]. Indeed, previous research has demonstrated that MS and chronic stress can impair NK cytotoxicity and hence tumor immunity [65]. In endometriosis, the impaired NK cell cytotoxicity as well as decreased NK cells in peritoneal fluid and sera have been noted since early 1990s [66–69]. Given the apparent elevated levels of anxiety and depression and the dysregulation of the HPA axis in mice exposed to MS, the presumed decrease in NK cytotoxicity would diminish the removal of endometriotic tissues, permitting their survival, implantation and proliferation. As such, the impaired NK cell function may also play a role in MS-accelerated lesional progression [70, 71].

ELA such as MS has been demonstrated to induce long-term changes in the central nervous system (CNS) in rodents, including those parts that modulate the integration of pain [72]. Rats exposed to MS are predisposed to develop visceral hyperalgesia, reduced somatic analgesia, and increased colonic motility in response to an acute psychological stressor, mimicking the cardinal features of irritable bowel syndrome [73]. The altered neuronal wiring and increased sensitivity to noticeptive stimuli would potentially exacerbate or intensify endometriosis-associated pain and thus further activate the HPA/SMA axes, forming another vicious cycle and thus promoting lesional progression. Our study has several strengths. First, we validated the finding that MS results in anxiety and depression-like behavior, which, in conjunction with lesional expression of ADRB2 and DRD2, provides evidence that HPA/SMA axes are involved. Second, we stained markers of EMT and FMT and also evaluated the extent of lesional fibrosis, which are known to be milestones of lesional progression [74].

Our study also has several limitations. First, we did not provide any mechanistic evidence as why MS accelerates endometriosis. This should await further investigations. Second, we only demonstrated MS instituted during PND 1–21 accelerates the progression of endometriosis. We did not identify which period in the infancy is the most critical or vulnerable, nor did we identify the minimal length of MS that is necessary to promote endometriosis progression. Future research is warranted to illuminate these issues. Hopefully, our study can pique the interest in this line of research, in particular in humans. Lastly, our study did not address a very important question as whether there may be ways to rectify the negative impact of ELA. Given that early MS led to anxiety and depression-like behavior in adulthood and that inflammation plays a critical role in depression [75, 76], one possible intervention might be to boost proresolving response through lipid mediators such as resolvins and lipoxin A4 [77]. Indeed, both resolvings and lipoxin A4 have been reported to suppress progression of endometriosis in rodent models [78–80]. Future studies are needed to further explore this line of research.

## Conclusions

In summary, this study demonstrates that exposure of female mouse pups to stress such as MS during their infancy period alters the activity of HPA axis and increases depression as well as anxiety levels in adulthood. More importantly, it demonstrates that ELA accelerates the progression of endometriosis, possibly through altered neuronal wiring and hyperactivity of the HPA axis.

## Supplementary information

**Additional file 1: Supplementary Figure S1**. Positive control and negative control for immunohistochemical staining. All magnifications: × 400. Scale bar = 50 μm.

## Data Availability

The dataset used in current study is available from the corresponding author on reasonable request.

## Abbreviations

α-SMA: α-smooth muscle actin
ADRB2: Adrenergic receptor β2
CREB: CAMP-response element binding protein
CNS: Central nervous system;
DOHaD: Developmental origin of adult health and disease
DRD2: Dopamine D2 receptor
ELA: Early-life adversity
EMT: Epithelial-mesenchymal transition
FMT: Fibroblast-to-myofibroblast transdifferentiation.
FST: Forced swimming test
HPA: Hypothalamic-pituitary-adrenal
MS: Maternal separation
MVD: Microvessel density
NK: Natural killer
NS: Non-separated
OFT: Open field test
PND: Postnatal day
SMA: Sympatho-adreno-medullary
SMC: Smooth muscle cell
SNS: Sympathetic nervous system
TST: Tail suspension test
